# Drop jump vertical kinetics identify male youth soccer players at greater risk of non-contact knee injury

**DOI:** 10.1016/j.ptsp.2025.03.003

**Published:** 2025-03-06

**Authors:** Jason S. Pedley, Rhodri S. Lloyd, Paul J. Read, Isabel S. Moore, Gregory D. Myer, Jon L. Oliver

**Affiliations:** a Youth Physical Development Centre, School of Sport and Health Sciences, Cardiff Metropolitan University, Cardiff, UK; b Sport Performance Research Institute, New Zealand (SPRINZ), AUT University, Auckland, New Zealand; c Institute for Sport, Exercise and Health, London, UK; d Centre for Sport Science and Human Performance, Waikato Institute of Technology, Hamilton, New Zealand; e School of Sport and Health Sciences, Cardiff Metropolitan University, Cardiff, UK; f Emory Sport Performance and Research Center, Flowery Branch, GA, USA; g Emory Sports Medicine Center, Atlanta, GA, USA; h Department of Orthopaedics, Emory University School of Medicine, Atlanta, GA, USA; i The Micheli Center for Sports Injury Prevention, Waltham, MA, USA

**Keywords:** Anterior cruciate ligament, Athletic injuries, Knee joint, Maturity, Stretch-shortening cycle

## Abstract

**Objectives:**

To determine associations between drop-jump vertical kinetics and acute non-contact knee injury-risk in male youth soccer players.

**Design:**

Prospective cohort study.

**Setting:**

Professional soccer academies.

**Participants:**

Youth soccer players (*n* = 264).

**Main outcome measures:**

Drop-jump vertical kinetics; injury epidemiology. Associations between kinetics and injury were assessed using binary logistic regression. Differences between injured and uninjured groups were compared using statistical parametric mapping.

**Results:**

Peak braking: peak propulsive force ratio (OR = 1.59, 1.10–2.29, *p* < 0.05), propulsive work (OR = 0.53, 0.28–0.99, *p* < 0.05) and vertical stiffness (OR = 1.68, 1.13–2.52, *p* < 0.05) were associated with risk of sustaining a knee injury. All variables demonstrated ‘unusable’ or ‘weak’ levels of predictive ability in identifying players who would become injured (AUC 0.568–0.663).

**Conclusions:**

Drop-jump vertical kinetics that characterise the shape of the force-time waveform provide insight to acute non-contact knee injury-risk in male youth soccer players. Large transient spikes in force in the early phase of ground contact, coupled with reduced propulsive forces are a risk factor for acute non-contact knee injury in male youth soccer players. Variables are not sensitive enough to predict injury but provide additional training targets to help mitigate risk in this population.

## 1. Introduction

The introduction of the Elite Player Performance Pathway (EPPP) by the English Premier League stipulated a year-on-year increase in training volume for academy players (Elite Player Performance Plan, 2011), and a rise in injury rates, particularly in the older age categories, has since followed (Tears et al., 2018). Chronological age and physical maturity do not progress in parallel; there are late maturing individuals undertaking increased training volumes despite still being relatively physically immature. Injury incidence rates show a rise in severe injuries (e.g. non-contact knee injuries) in male youth soccer players following peak height velocity (Monasterio et al., 2023), and such injuries can have long term effects on joint health (Shim et al., 2018). Understanding modifiable risk factors for these injuries enables the design of effective interventions to mitigate the risk of injury (Finch, 2006).

Drop jumps involve an impact, deceleration, and an immediate rebound jump which is similar to the mechanism of many non-contact knee injuries such as meniscal tears, medial collateral ligament tears and anterior cruciate ligament tears (Boden et al., 2000; Della Villa et al., 2020; Krosshaug et al., 2007; Pedley et al., 2017). Drop jump variables have previously been demonstrated to be associated with risk of knee injury risk in young soccer players (O’Kane et al., 2016). However, research into drop jumps as a screening tool has focused on the presence of medial translation of the knee in the frontal plane and other associated variables as an indicator of anterior cruciate ligament (ACL) injury risk (Pedley et al., 2020a). However, studies demonstrate that stiff landings are also a common aberrant movement pattern associated with non-contact knee injury including medial collateral ligament and meniscus tears (Krosshaug et al., 2007). Notably, injured participants have a greater likelihood to demonstrate lower hip and knee flexion at and during ground contact, along with greater peak vertical ground reaction force during a drop jump assessment (Krosshaug et al., 2007; Leppanen, Pasanen, Kujala, & et al, 2017). While evidence demonstrates greater prevalence of validated biomechanical risk factors in females (Smith et al., 2012), these risk factors are still associated with a greater risk of injury in male populations (Read et al., 2018b) albeit relatively unexplored in male youth, thus requiring further investigation.

Peak vertical ground reaction force has commonly been used to examine associations with injury during jump-landing tasks (Pedley et al., 2020a). However, it should be considered that the timing of traumatic knee injury such as an ACL rupture is usually within the first ~50 ms of ground contact when the knee is still in a high degree of extension (Krosshaug et al., 2007). In well executed drop jumps, peak force occurs much later than this at around 50% of ground contact time (~80–125 ms)(Pedley et al., 2020b-a). Peak force during this period is unlikely to have strong associations with acute non-contact knee injuries, however, peak force in less well executed drop jumps can occur much earlier as commonly observed in youth (Pedley et al., 2020b-a). In those with good stretch-shortening cycle (SSC) function peak force in a drop jump does not occur until long after ACL failure would have happened in a rebound task (Pedley et al., 2020b-a). Therefore, using peak vertical ground reaction force should also be contextualised as to when it happened during ground contact as this may display stronger associations with non-contact knee injuries commonly occurring in male youth soccer players (e.g. medial collateral ligament and meniscal tears), but this remains to be investigated.

Despite the limitations of peak vertical ground reaction force as an indicator of injury risk, it remains the only drop jump ground reaction force variable that has been analysed with regard to risk of injury (Pedley et al., 2020a). Force-time waveforms during a drop jump provide an indication of SSC function in young athletes (Pedley et al., 2020b-a), with a bell-shaped profile being indicative of spring mass model behaviour and enhanced SSC function. Conversely, transient spikes in force representing large loading rates in the early period of ground contact are indicative of worse engagement of feed-forward mechanisms such as pre-activation strategies and stretch reflexes (Bhattacharyya, 2017; Gollhofer et al., 1984), and are likely to occur as a result of inadequate force attenuation at the ankle joint (Gross & Nelson, 1988). This yielding of the ankle could increase the energy absorption requirements of the knee, particularly passive tissues such as ligament and bone, when the muscle-tendon unit is incapable of generating sufficient forces to absorb the energy (Krosshaug et al., 2007). In contrast, effective SSC function invokes pre-activation of muscles that stabilise joints of the lower limb and facilitates greater magnitudes of mechanical work at the knee, which might reduce the risk of acute non-contact knee injuries (Komi, 2000; Moran & Wallace, 2007).

Ratio of peak braking force to propulsive force has been observed to decline in males with advancing maturity but remained constant in females (Hewett et al., 2006; Pedley et al., 2020b, 2021-a). It was speculated that this might be associated with ACL injury risk since females experience a rise in ACL injury frequency in comparison to males following puberty (Hewett et al., 2006). While more recent research has shown small reductions in braking force to propulsive force ratio in post pubertal females, this was facilitated by increases in propulsive force rather than alteration of peak braking force (Pedley et al., 2021). Large peak braking forces, particularly early on during ground contact, are more indicative of a passive collision between the landing surface and the body rather than a controlled active deceleration of the body’s centre of mass and might expose individuals to a greater risk of acute knee injury (Nigg & Liu, 1999).

While the drop jump is considered the most utilised jump-landing screening assessment in prospective injury studies, study cohorts are heavily biased towards female participants and even fewer assess male youth (Pedley et al., 2020a). Asynchronous growth bone and soft tissue in youth and the potential effect of this upon injury risk and SSC function make translation of adult data to youth populations inappropriate (Malina et al., 2004). Recent evidence indicates that knee injuries present the greatest injury burden among male youth male soccer players (Wik et al., 2020) with most knee injuries resulting in moderate or severe time loss (Read et al., 2018a). Furthermore, youth soccer injury patterns are related to maturity status, with injury burden peaking during the pubertal growth spurt (Johnson et al., 2020) and older players sustaining more knee ligament sprains than their less mature peers (Wik et al., 2020). Nonetheless, there remains a paucity of data pertaining to injury risk factors in this population and the association between drop jump vertical kinetics and non-contact knee injury risk remains unexplored. Further, knee injury research has been largely focused upon ACL risk, though other structures in the knee are more commonly injured (Read et al., 2018a) and can be damaged through similar mechanisms and result in substantial time loss and long-term joint health issues (Boden et al., 2000). The aim of this study was to examine the relationship between drop jump vertical kinetics and the probability of sustaining any acute non-contact knee injury in academy youth soccer players. We hypothesised that force-time profiles characterized by large impact forces in the early part of ground contact and diminished force production in the propulsive phase would be associated with a greater likelihood of sustaining an acute non-contact knee injury in youth soccer players.

## 2. Methods

### 2.1. Participants and study design

**Fig. 1.**
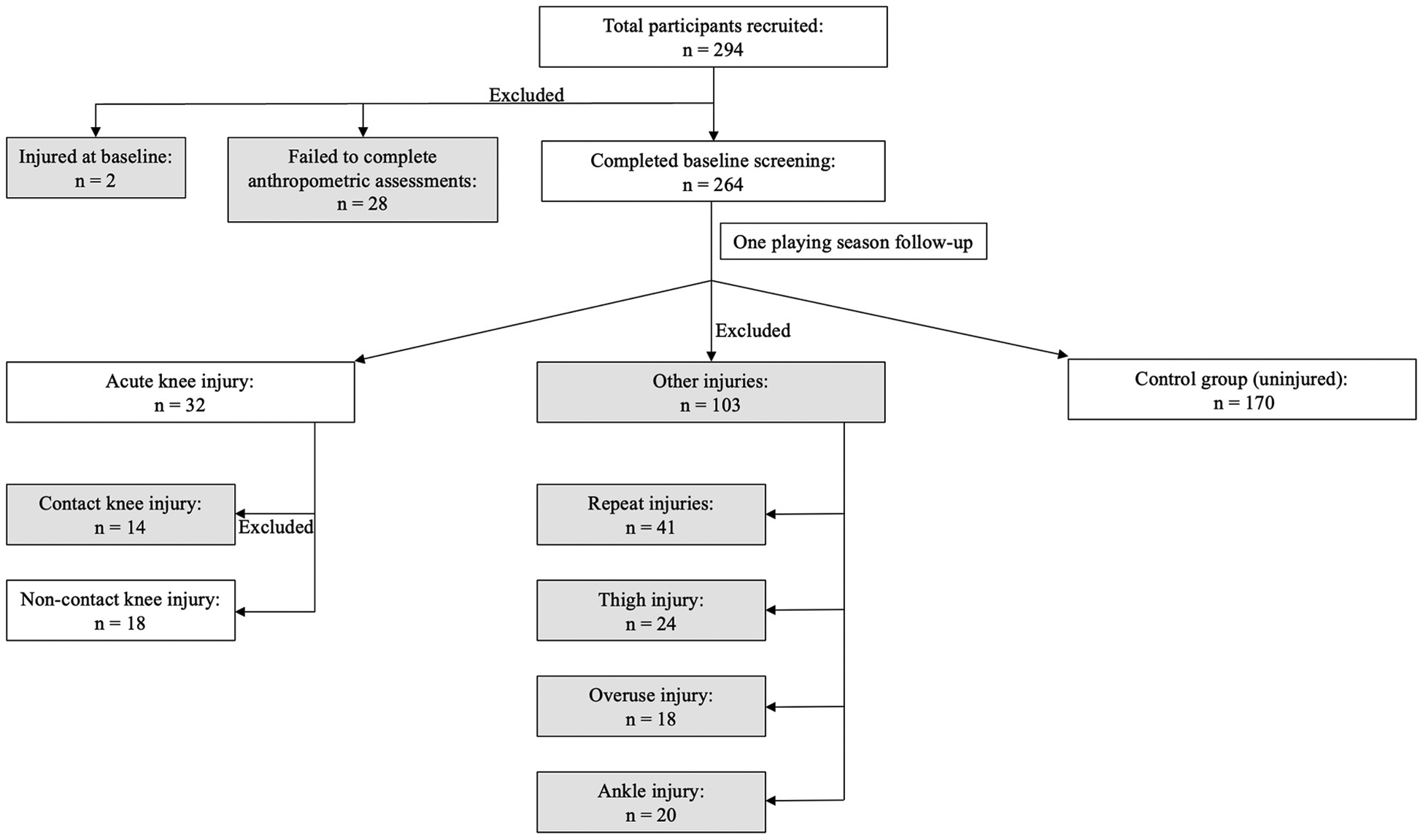
Flowchart illustrating recruitment of participants and subsequent inclusion and exclusion decisions resulting in the analysed study cohorts.

This prospective cohort study included 264 male youth soccer players enrolled in age group squads (under 11 to under 18s) within category 1 and 2 academies of professional English soccer clubs (Fig. 1). Sample size was calculated using peak vertical ground reaction force as a predictor and α = 0.05, power of 0.8, an injury rate of 20% and an odds ratio of 1.65 that would require a sample size of *n* = 205 (G*Power). This suggests the sample size was robust enough to tolerate typical dropout rates and still be able to address the research aim. Participants were required to be free from injury for 6 weeks prior to the commencement of the study and participating regularly in soccer training and matches in accordance with the requirements of the EPPP (under 11, 8 h/wk; under 12-under 16, 12–16 h/wk + strength and conditioning; under 17 – under 18, 16 h/wk + strength and conditioning). Any participants injured at the time of testing or during the 6 weeks prior were excluded from the study, as were any participants who were released by their club during the surveillance period or did not attend at least 80% of usual training and matches for any other reason. Goalkeepers were also not included in the study. A baseline drop jump assessment was conducted with proceeding injury surveillance. All participants were familiar with performing drop jumps as part of their habitual training routine. Cases were those soccer players who sustained an acute traumatic non-contact knee injury while playing or training for soccer. The control group were those players who did not sustain any injury throughout the surveillance period of one season.

### 2.2. Procedures

#### 2.2.1. Maturity assessment

Biological maturity was estimated using anthropometric measurements. Body mass (kg) was measured on a calibrated digital scale (Seca 875 Culta; Milan Italy) and standing height (cm) was recorded using an upright stadiometer (Seca 213). To determine sitting height and the subsequent calculation of leg length, the stadiometer was placed on top of a 60 cm box. The participants sat on the base of the stadiometer with their lumbar spine pressed against the upright element and the edge of the box sitting in the crease of the back of the knee. Sitting height (0.82 ± 0.08 m), standing height (1.62 ± 0.16 m), chronological age (age 13.95 ± 1.99 yrs) and body mass (52.27 ± 13.99 kg) were entered into a predictive equation developed by Mirwald et al. (Mirwald et al., 2002) to calculate a maturity offset (−0.19 ± 1.85 yrs), which estimates years from peak height velocity.


MaturityOffset(±0.59yrs)=-9.236+[0.0002708×LegLengthandSittingHeightInteraction]-[0.001663×AgeandLegLengthInteraction]+[0.007216×AgeandSittingHeightInteraction]+[0.02292×WeightbyHeightRatio]

### 2.3. Drop jump protocol

At the start of pre-season (July), all players completed a standardised progressive warm-up followed by a drop jump from a 30 cm box, onto force plates (PASPORT PS-2141, Pasco, Roseville, CA, USA) sampling at a frequency of 1000 Hz. Subjects were instructed to “step out onto an invisible box and drop directly downwards” (Pedley et al., 2017). Hands were kept on the hips while each foot was required to contact a separate force plate and the entirety of the contact area of the foot needed to be solely on the force plate rather than the wooden housing surrounding the platforms. Upon landing, subjects were instructed to “immediately jump as high as possible” and then stick the landing on the force plates. Failure to meet these criteria resulted in the trial being void. Two minutes of rest was observed between repetitions and jumps were repeated until three acceptable trials were collected for each participant. Participants performed the drop jump assessment in their own shoes.

### 2.4. Injury surveillance

All injuries experienced by participants during the soccer season were diagnosed and recorded by a medical professional at the club academy in accordance with the English Premier League EPPP regulations. An injury was defined as an incident that resulted in the player being unable to participate in training or match activities for 48 h or more from the day following the initial incident. Minor (3–7 days), moderate (8–28 days) and severe (>28 days) knee injuries occurring through non-contact or indirect contact mechanisms during soccer-related activity were of interest to the present study. Only the first knee injury for each participant was considered for the study, due to the confounding effect of previous injuries on subsequent injury risk.

### 2.5. Data processing

Kinetic data from the two force plates were combined and data from the first landing of the drop jump were analysed. Touchdown and toe-off of the first landing were defined as vertical force exceeding and then dropping below a threshold of 15 N respectively. A fourth order recursive low-pass Butterworth filter with a cut-off of 30 Hz was applied to vertical ground reaction force data using a customised MatLab programme (V.9.4.0.8, Natick, Massachusetts, USA). Calculated vertical kinetic variables and their definitions are provided in Table 1.

### 2.6. Statistical analyses

Kinetic variables were normalized to bodyweight and the mean of the three trials was used for statistical analysis. Independent samples *t*-tests with α = 0.05 were used to determine differences in discrete variables between the injured and uninjured groups. Associations between discrete kinetic variables and probability of sustaining an acute non-contact knee injury were assessed using binary logistic regression. Prior to running all regression models, the Box-Tidwell Test was used to check the assumption of linearity between the independent variables and their logit transformation. As there were too few injuries in maturity status sub-groups for between group comparisons, univariate binary logistic regression was performed with maturity offset entered as a covariate to account for the effects of maturation on ground reaction forces. All variables were converted into z-scores to produce odds ratios that were reflective of change in odds for a single standard deviation increase in the predictor variable while 95% confidence intervals were also calculated. Within-session reliability was determined using intraclass correlation coefficients with a two-way mixed effects model for average measures (ICC [3,k]).

Multivariate analysis was performed utilising only the variables that demonstrated *p* < 0.20 in the univariate analysis (Hewett et al., 2005). Variables with a variance inflation factor (VIF) > 10 were removed based on previous guidelines (Myers, 1990). This process was repeated with the variable with the next lowest clinical significance until all variables returned a VIF <10. The variables remaining, along with maturity offset, were then entered into the multivariate binary logistic regression model. Area under the receiver operator curve (AUC) was calculated to determine the predictive accuracy of each regression model in identifying participants who would subsequently suffer an acute non-contact knee injury. AUC of 0.50–0.59 were categorised as ‘unusable’, 0.60–0.69 as ‘weak’, 0.70–0.79 as ‘reasonable’ 0.80–0.89 as ‘good’ and 0.90–1.00 as ‘excellent’ predictive accuracy (Goossens et al., 2015). Cut-off values and their associated sensitivity and specificity for the independent variable were determined using Youden’s index. All statistical analyses were performed using Statistical Package for the Social Sciences (SPSS, v29.0.0.0. Chicago, Illinois).

Statistical parametric mapping was performed using MatLab (V.9.4.0.8, Natick, Massachusetts, USA) and open source package ‘spm1d’ (Pataky et al., 2013) used to identify periods of time during first ground contact where there were significant differences between the continuous time-normalized force-time waveforms of the injured and uninjured cohorts. A statistical parametric mapping (SPM) T-test was performed to calculate the scalar output statistics SPM{*t*} (Lohmander et al., 2004) at each individual time point to generate a statistical parametric map. Where the scalar statistic crossed the critical threshold (α = 0.05), the null hypothesis was rejected.

## 3. Results

During the entire playing season, 135 injuries were sustained by 94 players; 41 players sustained more than one injury, and 62 injuries were sustained to areas other than the knee. There were 32 acute knee injuries with 18 (age 13.94 ± 1.67 yrs; height 1.65 ± 0.15 m; 53.31 ± 13.57 kg; maturity offset −0.09 ± 1.69 yrs) occurring through non-contact or indirect contact mechanisms (Table 2). There were 170 players who did not sustain any injury throughout the season and served as the control group (age 13.96 ± 1.95 yrs; height 1.62 ± 0.14 m; 52.30 ± 13.10 kg; maturity offset −0.18 ± 1.71 yrs).

### 3.1. Differences in vertical kinetics between cohorts

**Fig. 2.**
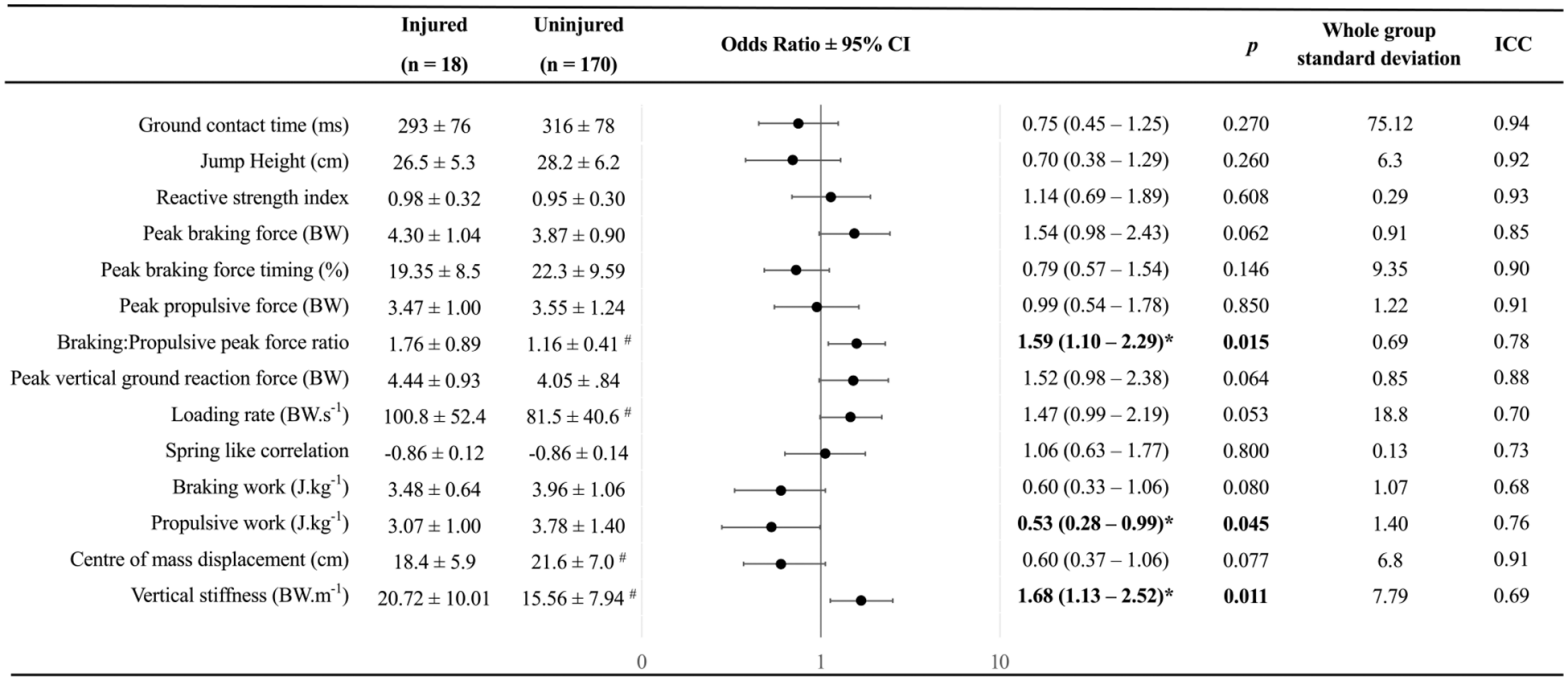
Between group T-test outcomes and univariate analysis of non-contact knee injury risk factors, controlled for maturity offset in elite youth soccer players. Intraclass correlation coefficients (ICC) for within-session reliability. Odds ratios with 95% confidence intervals are presented as change in risk per standard deviation (whole group). 1 = injury, 0 = no injury. # Significantly different to injured group (*p* < 0.05). * Significantly associated with odds of acute non-contact knee injury (*p* < 0.05).

**Fig. 3.**
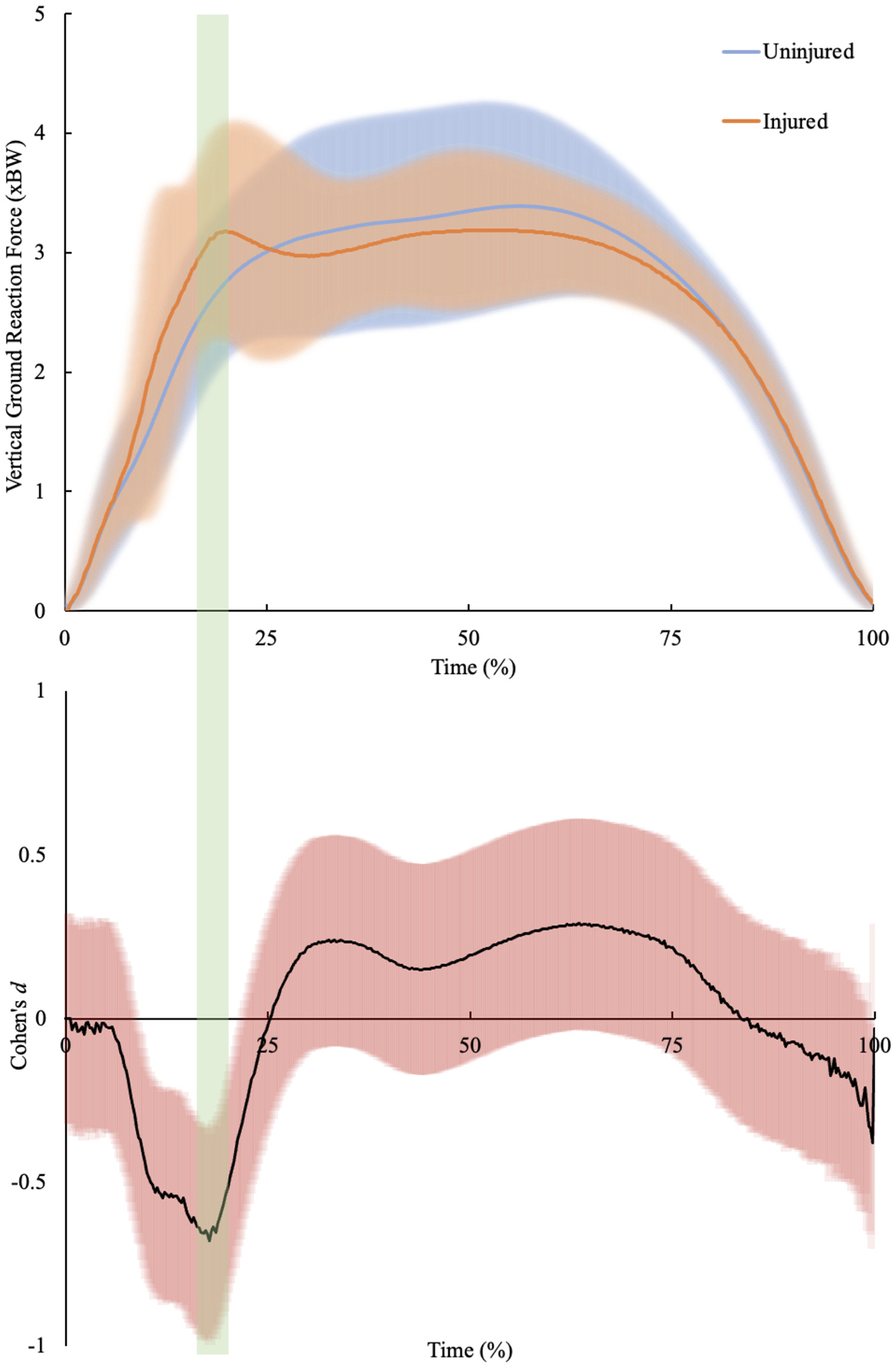
SPM *t*-test between time-normalized mean ground reaction force-time profile for injured and uninjured cohorts. Top-mean ± SD force-time waveforms. Bottom- Cohen’s *d* across ground contact period. Green shaded area indicates periods where there was a significant main effect between the two cohorts (*p* < 0.05).

Extraction of discrete variables from the force-time records showed that male youth soccer players who sustained an acute non-contact knee injury had greater braking: propulsive peak force ratio (Injured 1.76 ± 0.89 vs Uninjured 1.16 ± 0.41, *p* < 0.05, *d* = 0.87), greater loading rate (Injured 100.8 ± 52.4 BW.s^−1^ vs Uninjured 81.5 ± 40.6 BW.s^−1^, *p* < 0.05, *d* = 1.03), greater vertical stiffness (Injured 20.72 ± 10.01 BW. m^−1^ vs Uninjured 15.56 ± 7.94 BW.m^−1^, *p* < 0.05, *d* = 0.66) and lower centre of mass displacement (Injured 18.4 ± 5.9 cm vs Uninjured 21.6 ± 7.0 cm, *p* < 0.05, *d* = 0.47) (Fig. 2). Waveform analysis through statistical parametric mapping demonstrated differences in the force-time profiles between the injured and uninjured cohorts at 16–20% of ground contact time (*p* < 0.05, *d* = 0.46–0.68) (Fig. 3).

### 3.2. Univariate associations with knee injury risk

**Fig. 4.**
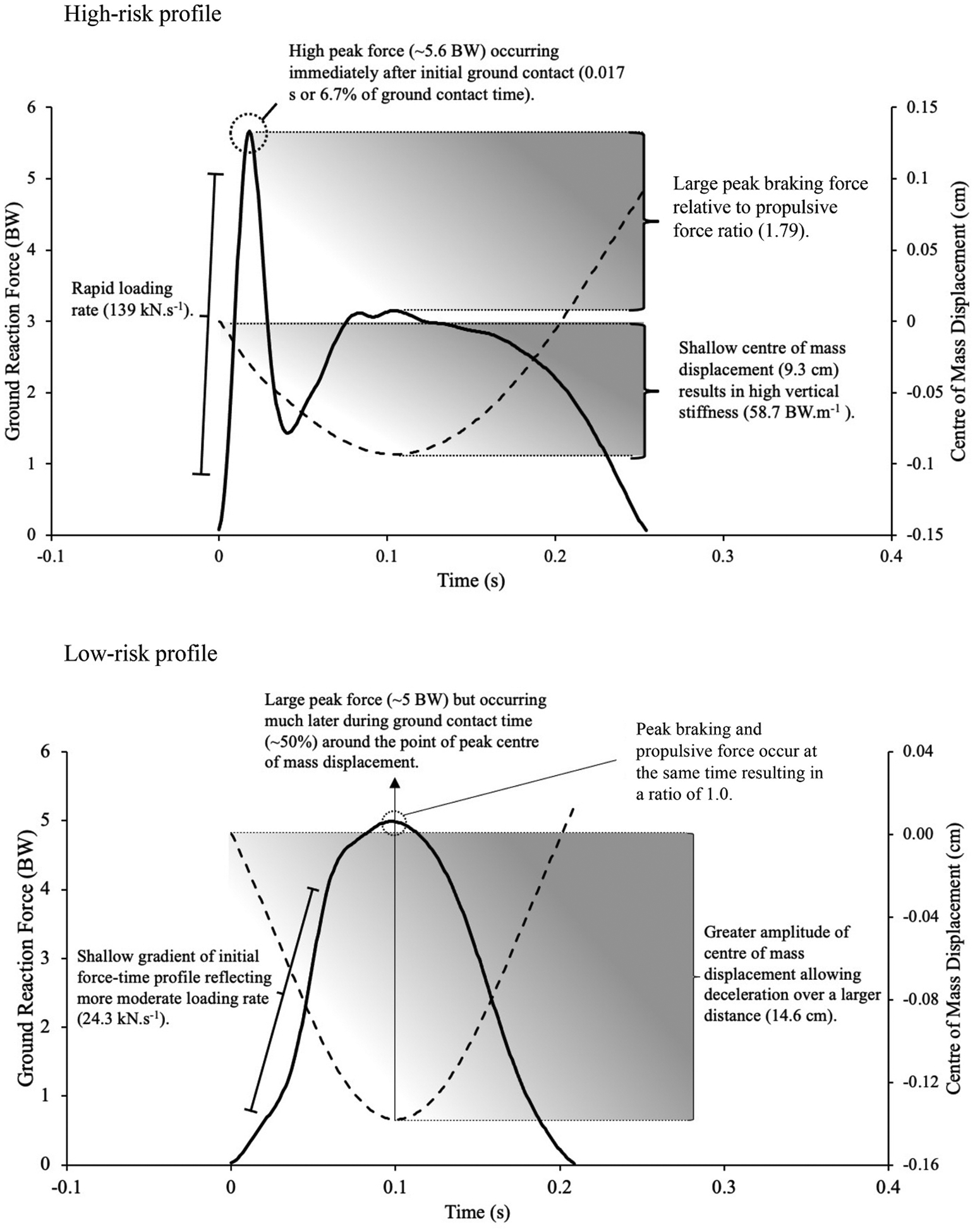
Example force-time profiles from the first landing of a drop jump for two contrasting athletes; black lines are vertical ground reaction force, dashed lines are vertical centre of mass displacement.

Three variables were significantly associated with risk of acute non-contact knee injury (Fig. 2). The risk of suffering a knee injury increased by 59% for each standard deviation (0.69) increase in ratio of peak braking force to propulsive force (OR = 1.59, 1.10–2.19). Furthermore, there was a 47% decrease in the probability of suffering a knee injury for each standard deviation (1.40 J kg^−1^) increase in propulsive work (OR = 0.53, 0.28–0.99). Increased vertical stiffness was associated with a 68% increase in risk of acute non-contact knee injury for each standard deviation (7.79 BW.m^−1^) increase (OR = 1.68, 1.13–2.52). The AUC analysis demonstrated “weak” or “unusable” predictive ability for all three variables (Table 3). While other associations were non-significant, a number approached significance including peak braking force (*p* = 0.062), peak vertical ground reaction force (*p* = 0.064), loading rate (*p* = 0.053), braking work (*p* = 0.080) and centre of mass displacement (*p* = 0.077). Fig. 4 presents example force-time profiles of a high and low risk individual.

### 3.3. Multivariate associations with knee injury risk

Nine variables presented *p* < 0.20 and were therefore eligible for input into the multivariate analysis. Checks for collinearity reduced this to a final list of braking: propulsive peak ratio, loading rate, braking work and vertical stiffness, which were entered into the multivariate analysis along with maturity offset as a covariate. When controlled for the other variables, only ratio of braking peak to propulsive peak presented a significant association with risk of suffering an acute non-contact knee injury (Table 4) with a single standard deviation (0.69) increase associated with a 63% greater risk of injury (OR = 1.63, 1.16–2.31). The logistic regression model displayed “reasonable” predictive ability (AUC = 0.748, *p* < 0.001) with a cut-off of *P* = 0.95749 achieving 94.4% sensitivity and 48.0% specificity.

## 4. Discussion

Our study identified significant associations between force-time variables and acute non-contact knee injury risk in male youth soccer players, supporting our hypothesis. We observed a high-risk profile characterised by a larger braking: propulsive peak force ratio, large peak braking force occurring in the very early stages of the ground contact phase resulting in a high loading rate, high vertical stiffness and reduced vertical displacement causing reduced mechanical work in the propulsive phase (Fig. 4). Given these findings, we accept our hypothesis and reject the null hypothesis, which proposed no associations between force-time characteristics and injury risk. These risk factors can be targeted as part of an integrated injury prevention framework.

Larger peak braking force relative to propulsive force were associated with a greater probability of sustaining an acute non-contact knee injury in male youth soccer players. Further, significant differences between the mean force-time waveforms of the two groups at the time of peak braking force for the injured group (16–20% of ground contact time) advocate for reporting peak force timing and magnitude within both the braking and propulsive phases of the first landing of a drop jump. This approach allows better understanding of the shape of the force-time waveform compared to a single peak force measure which appears to provide better understanding of performance and acute non-contact knee injury risk.

Following multivariate analysis, the ratio of peak braking force to peak propulsive force was the only variable associated with the risk of sustaining a non-contact knee injury. Hewett et al. (Hewett et al., 2006) previously measured ratio of peak braking force to propulsive force and observed that this declined in males with advancing maturity but remained constant in females. It was speculated that this might be associated with ACL injury risk since females experience a rise in ACL injury frequency in comparison to males following puberty. While more recent research has reported small reductions in braking force: propulsive force ratio in post pubertal females, this was facilitated by increases in propulsive force rather than alteration of peak braking force (Pedley et al., 2021). Large peak braking forces, particularly early on during ground contact (first 20% of ground contact time), are more indicative of a passive collision between the landing surface and the body rather than a controlled active deceleration of the body’s centre of mass (Nigg & Liu, 1999; Zadpoor & Nikooyan, 2011). This landing strategy appears to increase risk of acute non-contact knee injury in male youth soccer players, possibly through the redirection of force to passive tissues such as ligament rather than active tissue such as the muscle-tendon unit.

In the current study, greater vertical stiffness during landing was associated with an increased risk of knee injury for male youth soccer players. Previous literature has also demonstrated that stiff landings, involving large vertical ground reaction forces and reduced vertical displacement of the centre of mass are associated with greater risk of knee injuries (Leppanen et al., 2017a, 2017b). In contrast, Moran et al. (Moran & Wallace, 2007) demonstrated that good SSC function is dependent upon minimising vertical displacement of the centre of mass during ground contact. Our data demonstrated no significant difference in peak vertical ground reaction force, peak braking force or peak propulsive force between the injured and uninjured cohorts, but the injured participants underwent significantly less vertical displacement during ground contact. Drop jump force time profiles displaying large braking force peaks and relatively lower propulsive forces have been linked to poor SSC function (Pedley et al., 2020b, 2021-a). In the current study, the braking force: propulsive force peak ratio was significantly associated with a greater risk of acute non-contact knee injury in youth male soccer players. While large peak vertical ground reaction forces were observed in both the injured and uninjured group in the current study, and large peak forces have also been observed in individuals with both poor and good SSC function (Pedley et al., 2020b-a), it seems it is the distribution of force throughout the ground contact period which gives better insight into injury risk and perhaps also SSC performance. In the current study, waveform analysis illustrated differences in the shape of the force-time profile and when peak force occurs. Force-time profiles inclusive of large force peaks in the early phase of ground contact and lower peak propulsive force were associated with a greater risk of sustaining a knee injury. Consequently, ground reaction force testing and monitoring might provide a means of assessing both performance and injury risk for practitioners and thus offering clarity by aligning training objectives to mitigate injury risk and enhance performance. Importantly, while discrete ground reaction force variables offer insight to performance and injury risk beyond traditional drop jump metrics such as jump height and ground contact time, these should be interpreted within the context of the entire force-time profile.

In the present study, reduced mechanical work in the propulsive phase of the ground contact period was associated with an increased risk of male youth soccer players sustaining an acute non-contact knee injury during the season. Mechanically, reduced work is a result of less vertical displacement during landing. It is possible that athletes who are unable to generate high muscle forces to activate a rapid stretch of the tendon during rebound tasks such as the drop jump, experience a yielding at the active knee restraints. To decelerate quickly over a short distance, these athletes appear to utilise a strategy that places excessive force onto passive joint restraints (ligament and bone), which in combination with poor limb configurations at touchdown places them at a greater risk of injury. Previous literature highlights that more work performed at the knee joint is associated with better SSC function and drop jump performance (Moran & Wallace, 2007). It would appear that there is likely an individually determined optimal amount of knee work which directs strain away from passive structures and mitigates risk, while executing the braking phase quickly enough to maintain stretch on the series elastic component of the muscle-tendon unit and initiate the stretch reflex to optimise SSC performance (Pedley et al., 2017).

While significant associations were observed between some ground reaction force variables and the likelihood of sustaining an acute non-contact knee injury, the predictive accuracy of cut-off thresholds to optimise specificity and sensitivity were weak. Previous literature has highlighted the difficulty in predicting injuries due to the multifactorial nature of injury incidence (Bahr, 2016). Consequently, the findings of prospective injury studies such as ours are better applied in identifying risk factors and guiding the design of injury risk reduction programmes rather than for predicting which individuals will get injured. However, it should be acknowledged that we took the novel approach of attempting to identify risk factors for all acute non-contact knee injuries that are sustained through similar mechanisms. This contrasts with other studies that have looked at a single injury such as anterior cruciate ligament injury (Hewett et al., 2005). Our approach could reduce the false negatives from a diagnostic cut-off but might also increase the false positives. Ultimately, this would result in an overly cautious approach to quantifying risk which is likely preferable and has lower cost to the athlete.

Performance outcome measures such as jump height, ground contact time and reactive strength index have been shown to have no relationship with risk of lower extremity injury risk (Pedley et al., 2020a). Our study suggests this is also the case for non-contact knee injuries in male youth soccer players and given the observation that better performance outcomes can happen in the presence of poor SSC function (Pedley et al., 2020b-a), our findings highlight the value in performing more granular assessments of drop jump performance. The kinetic insight into SSC function can provide additional information to help design suitable holistic training interventions for youth athletes, especially given the influence of growth and maturation on SSC function (Pedley et al., 2020b-a).

Attentional focus studies demonstrate that encouraging male youth soccer players to focus on reducing ground contact time during a drop jump results in an increase in peak GRF, loading rate and impulse during the braking phase (Oliver et al., 2019-a). This is a common cueing strategy for coaches conducting plyometric training, however, the findings of the current study demonstrate that this could be underlining a high-risk landing strategy in individuals who already present large peak braking forces in the early stages of ground contact. Existing evidence demonstrates that while SSC function improves with maturation in male youth soccer players, some mature youths still display undesirable force-time profiles (Pedley et al., 2020b-a). For these athletes, alternative cueing strategies and training interventions might be more advantageous for short term risk mitigation and long-term physical adaptation.

Match and training exposure is a known contributor to the probability of sustaining an injury (Johnson et al., 2022). Training load was not monitored in the current study though minimum training volumes within each age group of soccer academies is stipulated by the EPPP to provide some standardisation (Elite Player Performance Plan, 2011). Additionally, the study cohort were youth soccer players and would have been experiencing growth throughout the surveillance period in addition to the physical stimulus of training. Only a single screening assessment was conducted with no control for time elapsed between the screening assessment and the injury. The population could have experienced training adaptations and would have undergone varying degrees of growth between baseline assessment and sustaining an injury. Most of the recorded injuries occurred within 120 days of the screening. Recent literature demonstrates modest increases in peak force values of 200–300 N between maturity groups differing in chronological age by ~2 years (Kumar et al., 2024). The data in the present study were normalized to bodyweight and maturity offset was entered into regression models as a covariate. Despite one injury occurring 234 days after screening, it is not likely that adequate growth would have taken place to significantly change outcome and function. Nonetheless, biomechanical changes between screening and injury cannot be ruled out and future research should utilise more frequent screening points to mitigate this limitation. However, this research design has been employed in previous literature (Goossens et al., 2015; Hewett et al., 2005; Leppanen et al., 2017a, 2017b; Read et al., 2018b) and this study is the first to present data assessing the utility of drop jump vertical kinetics for injury risk stratification in youth soccer players. Finally, only 18 acute non-contact knee injuries eligible for analysis were observed in the study. Previous studies have reported a knee injury incidence rate in this population of 20% (Read et al., 2018a) and we observed a first injury rate of 19%, though some of these injuries were excluded from the final analysis due to contact mechanisms. As a result, the risk of committing a type II error was elevated as a result of reduced statistical power.

## 5. Conclusion

Our findings demonstrated that while the magnitudes of peak force, peak braking force or peak propulsive force were not independently associated with probability of sustaining an acute non-contact knee injury, a larger ratio of peak braking force to peak propulsive force was associated with a greater likelihood of sustaining a knee injury during the season. Practitioners should avoid interpreting single measures of peak force without context of when that peak measure occurs and the appearance of the remaining force-time profile. The drop jump is a simple athletic task that requires little familiarisation or training history to perform. Force plates are now highly portable with many systems providing instant data processing to allow timely feedback to sports medicine practitioners and their athletes. Since drop jumps are a knee-dominant jump landing task (Hobara et al., 2009), ground reaction force profiling could be useful for practitioners to aid in the prescription and delivery of training interventions for male youth soccer players that target the reduction of potentially injurious landing strategies and develop an athlete’s SSC function. Further research is required to understand optimal training strategies for improving high risk injury profiles.

## Supplementary Material

1

## Figures and Tables

**Table 1:** Definitions of ground reaction force variables calculated during data processing.

Variable	Definition/calculation
Ground contact time (ms)	Time between first force value > 15 N and next force value < 15 N.
Jump height (cm)	Vertical displacement of the centre of mass during flight phase [(flight time)^2^ × (g/8)].
Reactive strength index	Ratio of jump height to ground contact time.
Centre of mass displacement (cm)	Peak vertical displacement of the centre of mass during ground contact time based on double integration with respect to time of the acceleration data derived from the vertical ground reaction force. This event is used to distinguish between the braking phase and the propulsive phase.
Peak braking force (BW)	Highest transient force peak during the braking phase of ground contact time. In the absence of a transient peak in the braking phase, the highest force value before peak centre of mass displacement is recorded.
Peak braking force timing (ms)	Timing of peak braking force.
Peak propulsive force (BW)	Highest transient force peak during the propulsive phase of ground contact time. In the absence of a transient peak in the propulsive phase, the highest force value after peak centre of mass displacement is recorded.
Braking: propulsive peak force ratio	Ratio of peak braking force to peak propulsive force
Peak vertical ground reaction force (BW)	Highest visible transient force peak during ground contact time.
Average loading rate (BW.s^−1^)	With initial ground contact as 0% and time of peak braking force as 100%, the gradient of the line between 20% and 80% of time to peak braking force was calculated.
Spring like correlation	Pearson product moment correlation between vertical ground reaction force and vertical displacement of centre of mass throughout ground contact time.
Braking work (J.kg^−1^)	Mechanical work performed by the centre of mass between initial ground contact and peak centre of mass displacement.
Propulsive work (J.kg^−1^)	Mechanical work performed by the centre of mass between peak centre of mass displacement and take-off.
Vertical stiffness (BW.m^−1^)	Ratio of peak force to centre of mass displacement at time of peak force during ground contact.

**Table 2:** Characteristics of acute non-contact knee injuries.

Injury severity	n	Days lost (Mean ± SD)	Injury burden (days)
Severe	10	41.0 ± 20.9	515
Moderate	5	15.0 ± 1.4	66
Minor	3	3.0 ± 1.4	12
Type of injury	n	Days from baseline (Median, min - max)	Injury burden (days)
Medial collateral ligament sprain	12	82, 13 – 234	390
Medial meniscal lesion	4	86, 32 – 120	94
Patellar subluxation	2	106, 62 – 151	109

**Table 3:** Predictive ability of ground reaction force variables significantly associated with increased risk of knee injury in male youth soccer players. ‘Cut-off’ is the value of the independent variable that maximised combined sensitivity and specificity on the receiver-operator curve.

	AUC	Cut-off	Sensitivity	Specificity	Accuracy
Braking: propulsive peak force ratio	0.651	1.415	42%	87%	84%
Propulsive work	0.568	4.25 J kg^−1^	90%	34%	38%
Vertical stiffness	0.663	4.5 BW.m^−1^	5%	99%	93%

AUC- Area under receiver operator curve; BW- bodyweight;

**Table 4:** Multivariate analysis of knee injury risk factors. Variables were inputted as z-scores to generate odds ratios reflective of the increased odds of injury per one standard deviation increase in the independent variable.

	β	Odds ratio (95% CI)	*p*	Group mean ± SD
Braking: propulsive peak ratio (Z)	−0.491	1.63 (1.16–2.31)*	0.005	1.20 ± 0.69
Loading rate (Z)	0.197	0.82 (0.34–1.96)	0.658	82.8 ± 41.7 BW.s^−1^
Braking work (Z)	−0.237	1.27 (0.56–2.89)	0.573	3.93 ± 1.07 J kg^−1^
Vertical stiffness (Z)	−0.622	1.86 (0.64–5.46)	0.257	15.91 ± 7.79 BW.m^−1^
Maturity offset (Z)	−0.305	1.36 (0.76–2.41)	0.298	−0.19 ± 1.85 yrs
Constant	−2.922			

BW- bodyweight; SD— standard deviation

* *p* < 0.05

## Appendix A. Supplementary data

Supplementary data to this article can be found online at https://doi.org/10.1016/j.ptsp.2025.03.003.
